# A Nationwide Chronic Disease Management Solution via Clinical Decision Support Services: Software Development and Real-Life Implementation Report

**DOI:** 10.2196/49986

**Published:** 2024-01-19

**Authors:** Mustafa Mahir Ulgu, Gokce Banu Laleci Erturkmen, Mustafa Yuksel, Tuncay Namli, Şenan Postacı, Mert Gencturk, Yildiray Kabak, A Anil Sinaci, Suat Gonul, Asuman Dogac, Zübeyde Özkan Altunay, Banu Ekinci, Sahin Aydin, Suayip Birinci

**Affiliations:** 1 Ministry of Health Turkey Ankara Turkey; 2 Software Research Development and Consultancy Corporation Ankara Turkey

**Keywords:** chronic disease management, clinical decision support services, integrated care, interoperability, evidence-based medicine, medicine, disease management, management, implementation, decision support, clinical decision, support, chronic disease, physician-centered, risk assessment, tracking, diagnosis

## Abstract

**Background:**

The increasing population of older adults has led to a rise in the demand for health care services, with chronic diseases being a major burden. Person-centered integrated care is required to address these challenges; hence, the Turkish Ministry of Health has initiated strategies to implement an integrated health care model for chronic disease management. We aim to present the design, development, nationwide implementation, and initial performance results of the national Disease Management Platform (DMP).

**Objective:**

This paper’s objective is to present the design decisions taken and technical solutions provided to ensure successful nationwide implementation by addressing several challenges, including interoperability with existing IT systems, integration with clinical workflow, enabling transition of care, ease of use by health care professionals, scalability, high performance, and adaptability.

**Methods:**

The DMP is implemented as an integrated care solution that heavily uses clinical decision support services to coordinate effective screening and management of chronic diseases in adherence to evidence-based clinical guidelines and, hence, to increase the quality of health care delivery. The DMP is designed and implemented to be easily integrated with the existing regional and national health IT systems via conformance to international health IT standards, such as Health Level Seven Fast Healthcare Interoperability Resources. A repeatable cocreation strategy has been used to design and develop new disease modules to ensure extensibility while ensuring ease of use and seamless integration into the regular clinical workflow during patient encounters. The DMP is horizontally scalable in case of high load to ensure high performance.

**Results:**

As of September 2023, the DMP has been used by 25,568 health professionals to perform 73,715,269 encounters for 16,058,904 unique citizens. It has been used to screen and monitor chronic diseases such as obesity, cardiovascular risk, diabetes, and hypertension, resulting in the diagnosis of 3,545,573 patients with obesity, 534,423 patients with high cardiovascular risk, 490,346 patients with diabetes, and 144,768 patients with hypertension.

**Conclusions:**

It has been demonstrated that the platform can scale horizontally and efficiently provides services to thousands of family medicine practitioners without performance problems. The system seamlessly interoperates with existing health IT solutions and runs as a part of the clinical workflow of physicians at the point of care. By automatically accessing and processing patient data from various sources to provide personalized care plan guidance, it maximizes the effect of evidence-based decision support services by seamless integration with point-of-care electronic health record systems. As the system is built on international code systems and standards, adaptation and deployment to additional regional and national settings become easily possible. The nationwide DMP as an integrated care solution has been operational since January 2020, coordinating effective screening and management of chronic diseases in adherence to evidence-based clinical guidelines.

## Introduction

As in the rest of the world, the aging population is increasing rapidly in Turkey. A recent TurkStat report predicts that by 2030, the older adult population will be 12.9%, rising to 22.6% in 2060 and 25.6% in 2080 [1]. Noncommunicable diseases are the leading cause of death and disability in Turkey, posing a significant burden [2]. The elevated health costs for older adults strain Turkey’s health care system. To address this, the Turkish Ministry of Health (MOH) has implemented a national strategy emphasizing multidisciplinary teams, led by family physicians. The goal is to enhance early detection and manage complications of noncommunicable diseases through systematic screening programs under the national Disease Management Platform (DMP) project launched in late 2018.

The growing use of digital health solutions such as electronic health records (EHRs) presents an opportunity to enhance chronic disease management. Clinical decision support services (CDSSs) can assist in making patient-centered and evidence-based decisions [3,4]. Digital tools and systems that collect and use patient information to provide decision support for health care professionals (HCPs), including patient-specific assessments and recommendations, can promote adherence to national guidelines, ultimately resulting in enhanced quality of care [5-9]. Research demonstrated that computerized decision support tailored to the patient successfully improved decision-making [10,11]. Such tools enhanced the decision-making abilities of HCPs in various domains, including effective prescription decisions [12,13], adherence to guidelines for cardiac rehabilitation [14], management of hypertension and diabetes [15-21], cancer screening [22,23], and computerized order decisions [24,25].

Building on these results, the national DMP is designed as an integrated care platform for chronic disease management in Turkey in a family physician–centered manner. It aims to effectively implement clinical treatment protocols, ensuring easy adherence with decision support services. These services focus on early diagnosis, followed by structured treatment recommendations during routine follow-ups. The DMP enhances standardization of care, improving health care efficiency and quality. It also facilitates seamless transitions between primary care and specialist services, reducing costs, minimizing risks, eliminating redundant tests, and easing the burden on patients.

To ensure successful implementation of a DMP aimed at achieving these strategic objectives, several technical challenges need to be addressed. Our design decisions consider the crucial factor of integrating CDSSs seamlessly into clinicians’ daily workflow [26,27]. Despite the potential of CDSSs for evidence-based medicine, significant effort is needed to realize these benefits [28]. The DMP must smoothly integrate with physicians’ workflows, necessitating interoperability with existing health IT systems. CDSS guidance should be user-friendly, ensuring a natural flow for clinical protocol implementation. With a target audience of over 26,000 practitioners in Turkey, serving a population of over 85 million, the platform must ensure high performance and scalability. It should easily expand to address additional diseases within a reasonable timeframe and prioritize reusability and compliance with international health IT standards for versatile deployment.

This paper outlines the design, development, nationwide implementation, and initial performance results of the national DMP in Turkey. The DMP can be categorized as a “2.3-Healthcare Provider Decision Support System” in terms of World Health Organization “Classification of digital health interventions” [29]. This implementation report will focus on the results of the deployment and implementation of the DMP in Turkey serving to more than 26,000 family medicine practitioners (FMPs) in the country. The objective is to share our experiences in building the DMP, as an implementation report in line with *iCHECK-DH: Guidelines and Checklist for the Reporting on Digital Health Implementations* [30]. We detail the design decisions and technical solutions aimed at ensuring interoperability with existing IT systems, integration with clinical workflow, enabling smooth transition of care, user-friendliness for HCPs, scalability, and adaptability in the *Methods* section. The *Implementation (Results)* section presents the outcomes of the nationwide implementation (number of users, number of screening and monitoring encounters, number of patients covered via these encounters, number of patients diagnosed as a result of screening encounters, and treatment goal achievements [such as blood pressure targets, hemoglobin A_1c_ {HbA_1c_}, and cholesterol targets]), demonstrating how these objectives were achieved. Additionally, we outline current limitations and identify areas for future work to further enhance the clinical impact.

## Methods

### Overall System Architecture and Design Decisions

**Figure 1:**
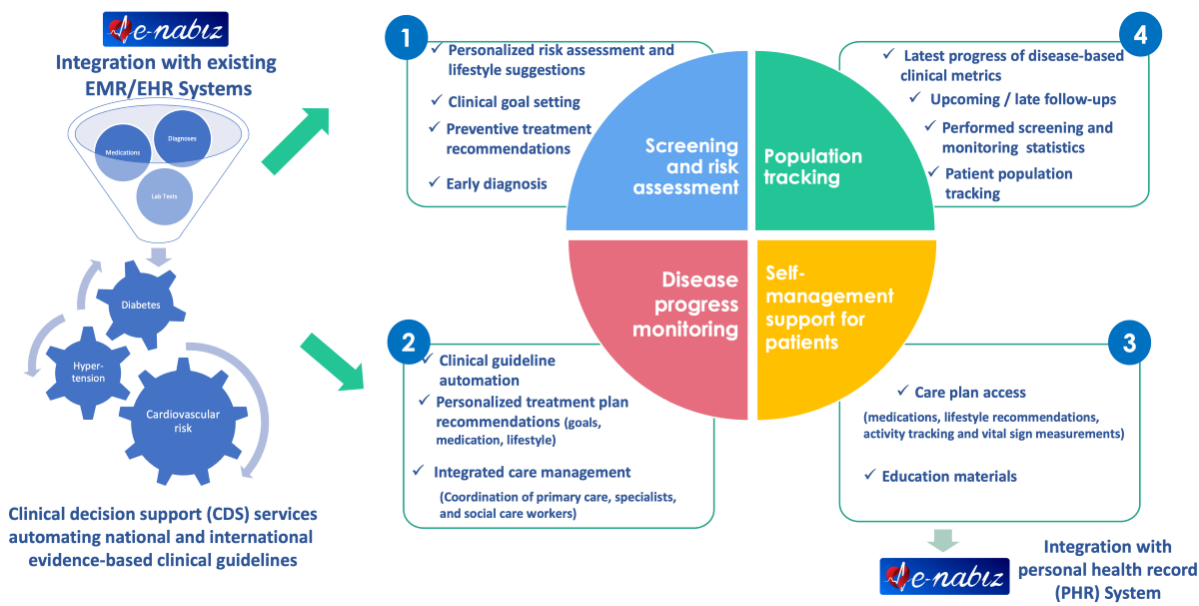
Overall aims of the disease management platform architecture. EHR: electronic health record; EMR: electronic medical record.

The DMP has been designed and implemented to enable the following 4 high-level features as summarized in Figure 1:

- Screening and risk assessment for healthy population: a web-based platform for FMPs facilitates screening for the healthy population. For instance, diabetes screening is required every 3 years for citizens aged over 40 years without a diabetes diagnosis. The full eligibility criteria for both screening and monitoring are presented in Multimedia Appendix 1. The system offers personalized risk assessments, early diagnosis, individualized goals, preventive treatment, and lifestyle suggestions aligned with national care pathways. Diagnosed patients enter the disease progress monitoring program, whereas undiagnosed individuals receive intensified screening based on risk and lifestyle recommendations.
- Disease progress monitoring: for diagnosed patients, the platform facilitates creating and updating personalized care plans during regular follow-up encounters, aligning with evidence-based national care pathways. It assesses laboratory results, conducts risk assessments, recommends personalized treatment goals and medications, suggests follow-up appointments, and refers to specialists when necessary for consultations and complication management. Patients in the monitoring program are categorized based on their control of clinical parameters, symptoms, and goal achievement status, guiding decisions on follow-up frequency, secondary care referrals, and medication plan updates.
- Self-management support for patients: a care plan with instructions for FMPs, specialists, and patients is shared with Turkey’s e-Nabiz platform, the national EHR and personal health record (PHR) system. Patients can then access details about care plan activities, including medications, educational materials, self-measurement activities, and lifestyle recommendations.
- Population tracking: each FMP manages 2000 to 4000 patients based on their region’s population. The population tracking module allows them to filter and manage patients for upcoming or overdue screening and monitoring encounters, access statistics on the screened population, send SMS invitations to patients, and monitor goal achievement for clinical parameters such as fasting plasma glucose, HbA_1c_, and blood pressure.

**Figure 2:**
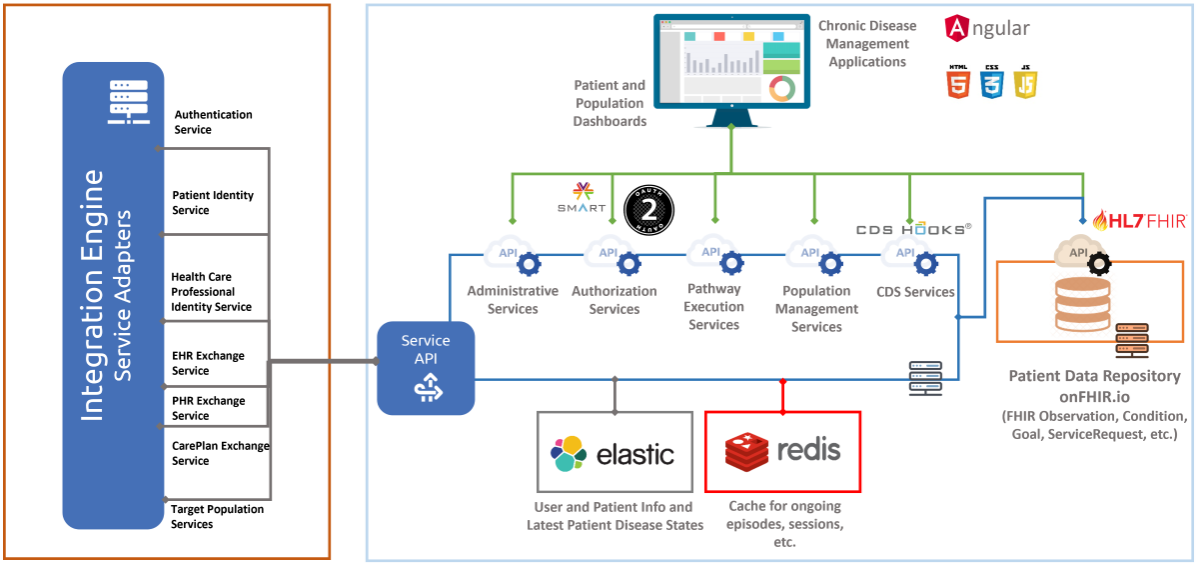
High-level system architecture of the disease management platform. API: application programming interface; CDS: clinical decision support; EHR: electronic health record; FHIR: Fast Healthcare Interoperability Resources; HL7: Health Level Seven; PHR: personal health record.

The overall system architecture of the DMP is depicted in Figure 2.

### Seamless Integration and Interoperability With Existing Systems

The DMP is designed and implemented for seamless integration with existing regional and national health IT systems. To achieve this, we have designed the core data model and data processing architecture of the DMP based on Health Level Seven (HL7) Fast Healthcare Interoperability Resources (FHIR) Release 4 [31]. FHIR has gained widespread adoption in the health care industry [32-36] and endorsed by country-wide implementations in the United States [34], United Kingdom [37], and Germany [38].

The DMP core data model conforms to HL7 FHIR Release 4 to encompass basic EHR components as well as resources for representing a patient’s care plan. An open-source HL7 FHIR Repository, namely onFHIR.io [39], serves as the main component of the data management layer (Figure 2). onFHIR.io uses MongoDB as a database and provides real-time data subscription with the help of Apache Kafka. The DMP web application directly accesses patient and care plan data through RESTful interfaces provided by onFHIR.io, enabling fine-grained access control over all FHIR resources in compliance with the SMART on FHIR authorization guidelines and scopes [40].

In Turkey, the MOH operates e-Nabiz, a central national EHR infrastructure [41]. This system collects patient records as encounter summaries from nationwide health care providers, with patients also inputting vital signs and activity data. e-Nabiz codes data using international and national medical terminology, such as *International Classification of Diseases, Tenth Revision* (ICD-10). It is a document-based repository accessed through a Representational State Transfer application programming interface [42], and interoperability adapters in the DMP project (EHR exchange and PHR exchange services in Figure 2) communicate with it to retrieve patient data. These adapters transform proprietary XML formats to HL7 FHIR-based data models and store them in the Patient Data Repository. This transformation includes both structural and semantic mapping, incorporating a strategy of incremental synchronization. On initial DMP access, the patient’s longitudinal EHR is mapped to FHIR, and subsequent encounters retrieve and transform only new, unsynchronized data.

To secure patient data access, clinicians authenticate to the DMP through the MOH’s central authentication and authorization services using the OpenID Connect protocol. The DMP uses a role-based access control mechanism, catering to different disease management roles. Before data access and synchronization, a check ensures that the user has the required access rights via the MOH’s central authentication service. If authorized, the DMP generates a patient-specific JavaScript Object Notation Web Token with corresponding permissions, serving as an OAuth2.0 bearer token for all interactions within the DMP.

In the DMP, FMPs perform screening and monitoring encounters based on predefined eligibility criteria. For instance, hypertension monitoring is required every 3 months for patients with a hypertension diagnosis and on antihypertensive medications. These criteria are executed in the e-Nabiz data warehouse, and both DMP and family medicine information systems retrieve target population lists through target population services (Figure 2). FMPs can easily identify if a visiting patient is on the screening or monitoring list via family medicine information systems, initiating a DMP encounter directly with a single sign-on integration.

The care plan created with the help of the DMP is stored as an HL7 FHIR *CarePlan* resource in the Patient Data Repository. It is shared with the e-Nabiz system via the Care Plan Exchange Service (Figure 2), enabling it to be accessible to the patient via e-Nabiz interfaces.

The DMP uses Elasticsearch technology for storing user information, basic patient attributes, and their current screening and monitoring statuses for each disease. Elasticsearch also serves as a system log repository. We have developed a Kibana interface for monitoring system performance and geographical statistics. Redis is used as a caching system to temporally store information about ongoing encounters and user authorization access tokens.

### Automation of National Care Pathways as a Clinical Workflow for FMPs

The interfaces of the DMP have been designed with ease of use in mind to allow for seamless integration into the regular clinical workflow. It is implemented as a cocreation activity with the involvement of system analysts, software engineers, and a clinical reference group set up by the MOH Department of Chronic Diseases and Elderly Health including multidisciplinary HCPs.

The national evidence-based care pathways have been collaboratively analyzed, leading to the identification of common steps, such as physical examination, medical history review, risk assessment, medication review, lab results review, diagnosis, clinical goal setting, pharmacological treatment planning, and nonpharmacological treatment planning. Each care pathway is designed modularly within the DMP as a series of pages corresponding to these common steps. These are organized as a flow of pages that is followed automatically based on patient parameters.

Each page is meticulously designed, specifying patient parameters for assessment. Most data come from the national EHR system, enabling clinicians to review prefilled pages with the latest parameters and make adjustments as needed. Validity periods for each parameter are identified, emphasizing recency, and they are enforced by the system and reminded to FMPs. Additionally, scaled assessments (eg, Mini Nutritional Assessment), risk assessments (eg, cardiovascular risk), and associated algorithms (eg, SCORE-Turkey) are also identified. Business rules within the pages are designed for personalized suggestions aligned with evidence-based care pathways.

**Figure 3:**
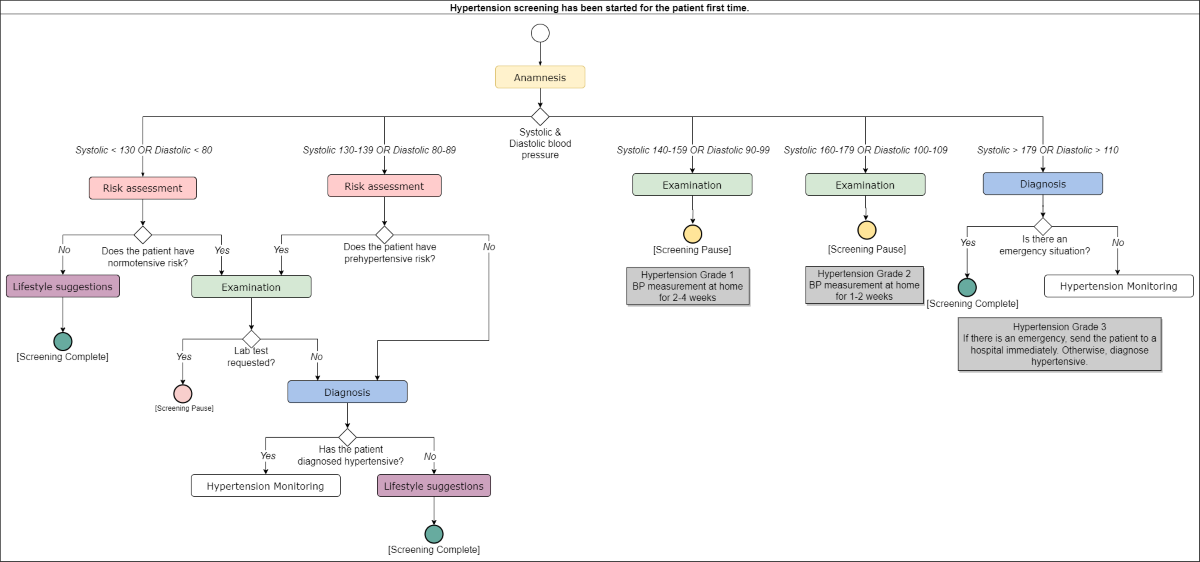
Hypertension screening flow. BP: blood pressure.

All of these are thoroughly documented after discussions in cocreation workshops. Mock-up screens are designed, and flow diagrams are created to identify transition criteria between pages. These materials undergo further review and finalization in subsequent cocreation workshops. As an illustrative example, Figure 3 depicts a sample flow for hypertension screening.

After cocreation, each step’s design becomes a web-based interface in the DMP application, developed with the Angular framework. A “Pathway Execution Service” state manager automates the flow diagram for disease screening or monitoring, adapting to patient parameters. This allows FMPs to use a wizard-like interface for encounters, facilitating adherence to national clinical pathways.

Transitions between disease modules are also modeled and implemented. For example, in hypertension screening, if a patient’s fasting plasma glucose exceeds 110 mg/dL, the system prompts FMPs to consider a diabetes screening if not already monitored for diabetes. In response, the patient’s diabetes screening schedule is automatically updated.

### CDSS Implementation

**Figure 4:**
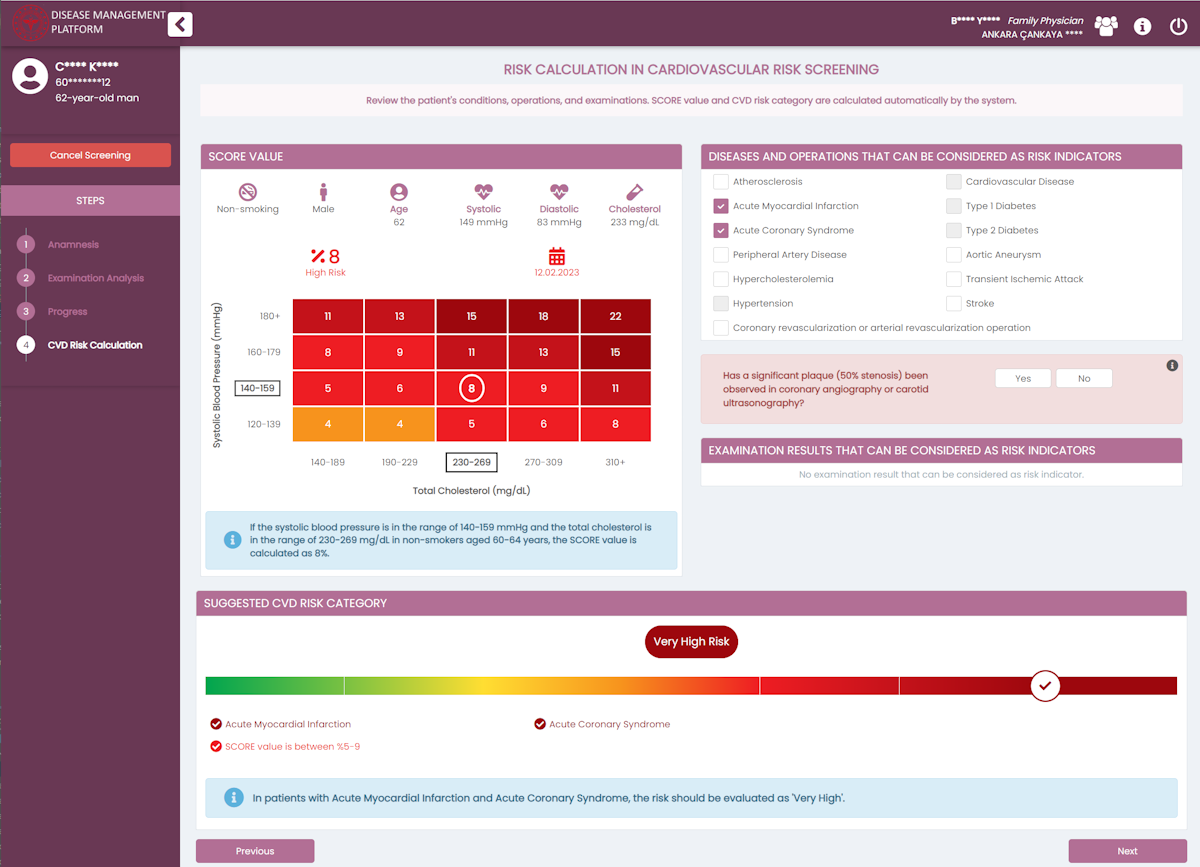
An example screenshot from the Cardiovascular Risk Screening Module presenting individualized risk calculation. (The system is implemented in a multilingual manner supporting Turkish and English by default.) CVD: cardiovascular disease.

**Figure 5:**
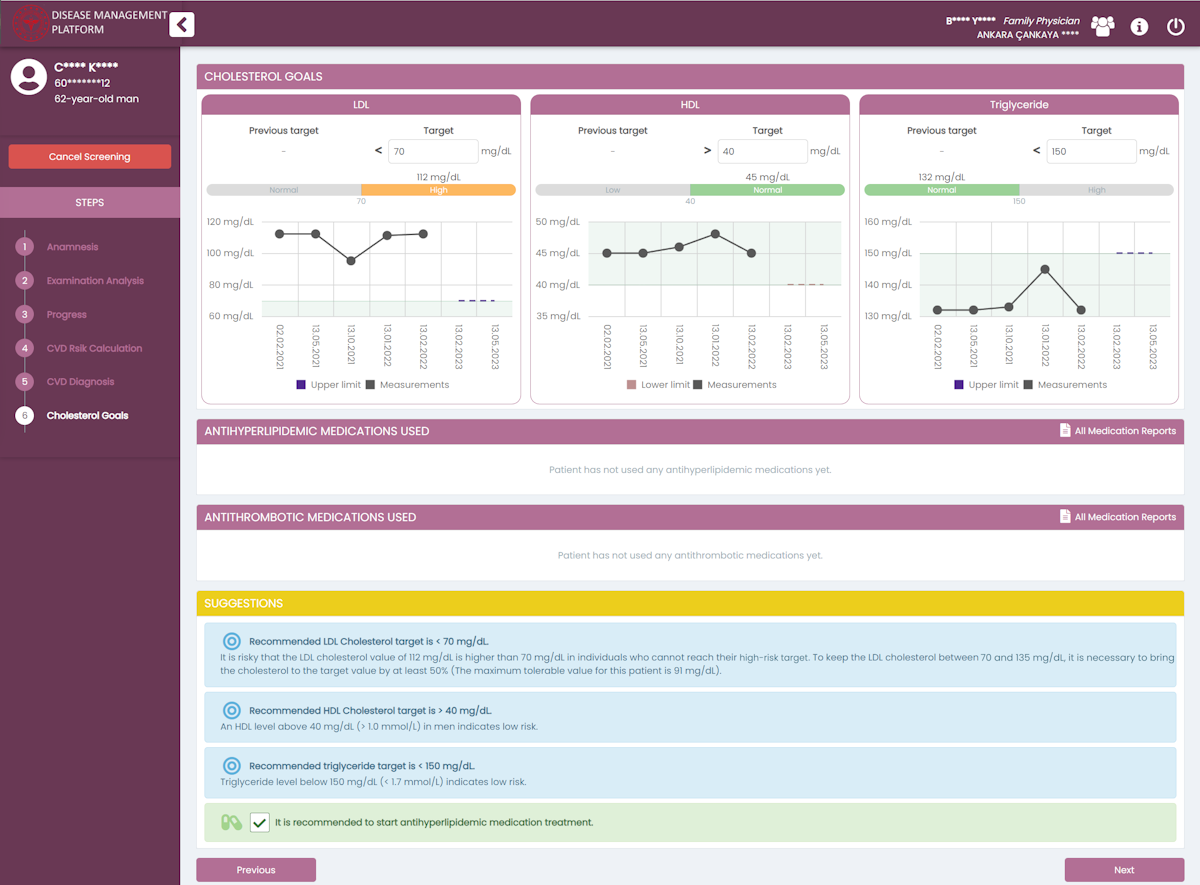
An example screenshot from the disease management platform presenting personalized lipid goals for the patient. HDL: high-density lipoprotein; LDL: low-density lipoprotein.

CDSSs are a core component of DMP to enable patient-tailored recommendations. On the basis of the documented business rules from the design phase, we have designed CDSSs as automated processes. These processes link patient-specific data with evidence-based knowledge from national care pathways. We can categorize the CDSS implemented based on their functionality as follows:

- Risk assessment via scored algorithms (eg, SCORE-Turkey): FMPs are provided with explanatory guidance about scoring, referencing validated scoring assessment algorithms (see Figure 4).
- Diagnosis recommendations based on the patient’s current condition and risk assessment: in screening operations, the CDSS recommends diagnoses to FMPs using predefined ICD-10 codes.
- Guidance for lab test ordering and interpretation: a personalized list of required lab tests is determined based on the patient’s disease state, risks, and other comorbidities. The CDSS also provides notifications for when these lab tests should be renewed on expiration.
- Diagnosis and referral suggestions are recommended based on patient parameters such as lab results. For example, referral to a nephrologist is recommended when the estimated glomerular filtration rate result is below 60 mL/min/1.73 m^2^.
- Treatment goals (eg, low-density lipoprotein cholesterol) are recommended based on the patient’s risk, disease stage, and comorbidities. In Figure 5, an example screen for goal planning is presented. The physician can always manually update these targets based on their assessments.
- Medication suggestions are recommended for treatment planning based on disease stage, response to previous medications, existing medications, and comorbidities. Certain medications are marked as contraindications based on the existing comorbidities of the patient.
- Referral suggestions for preventive consultation visits are recommended, especially for complication management. For instance, a yearly retinopathy check with an ophthalmologist is advised during diabetes monitoring encounters.
- Follow-up visits are recommended based on the current status of the patient. For instance, screening in each 2 years is suggested for patients with low cardiovascular disease risk, whereas once a year screening is suggested for high-risk patients.
- Automated care pathway transitions for patients with multiple morbidities are personalized based on specific disease criteria. For instance, if a patient aged over 40 years has not had their cardiovascular risk score calculated, the DMP guides FMPs to continue with the cardiovascular risk module during hypertension or diabetes monitoring.

In the DMP, all CDSS implementations adhere to the CDS Hooks specifications [43]. As a standard published by HL7, it provides an API specification for CDS calls. Both input parameters and output suggestions are defined in reference to HL7 FHIR resources, facilitating plug-and-play interoperability with platforms that support HL7 FHIR. The CDS Hooks–compliant approach allows easy expansion with CDSSs created by external entities and to simplify deployment in different settings already adhering to HL7 FHIR.

### Ensuring Performance of the System

The DMP is designed for high horizontal scalability, using 2 servers for the web application and 3 for the Patient Data Repository, forming an onFHIR.io server cluster. Nginx acts as both a reverse proxy and a load balancer to distribute traffic across these backend servers. onFHIR.io servers connect to a horizontally scalable MongoDB cluster for data distribution and replication. Elasticsearch log and data store operate on a cluster hosted on 6 servers.

### Testing, Piloting, and Deployment of Disease Modules

The system is developed by SRDC on behalf of the Turkish MOH with the support of Türksat and Innova. The final product is owned by the Turkish MOH. The initial version of the DMP, including modules for screening and monitoring of type 2 diabetes, hypertension, and cardiovascular risk management, was extensively tested by the clinical reference group. It underwent a 3-month pilot phase in 4 cities in late 2019. The pilot phase involved 14,351 encounters conducted by 219 FMPs for 5521 patients. Two more modules for obesity screening and monitoring, as well as older adult monitoring, were added to the system during this period. After the feedback is addressed and the system is retested, the system has been operationalized in whole Turkey by January 2020. On June 30, 2021, the MOH has published a directive incentivizing FMPs to conduct screening and monitoring for diabetes, hypertension, cardiovascular disease risk management, obesity, and older adult monitoring via the DMP. The system with incentivization calculations has been operational in whole Turkey since July 1, 2021.

Beyond existing modules, the system now includes monitoring modules for coronary artery disease, chronic kidney disease, stroke, chronic obstructive pulmonary disease, and asthma. The cocreation process, covering requirement analysis, mock-up design, implementation, and testing, took 3 months for each module, showcasing the process’s repeatability for swift module additions. These new disease modules are not yet public in the operational system.

## Implementation (Results)

The system is being used extensively throughout the whole country. As of September 18, 2023, a total of 73,715,269 screening and monitoring encounters have been performed by 25,568 users (24,627 FMPs and 941 FMP nurses) for 16,058,904 unique citizens. Among these citizens, 56.2% (n=9,025,104) are female and 43.8% (n=7,033,800) are male. The average number of DMP encounters per patient is 4.59. The distribution of encounters per DMP module and the breakdown between screening and monitoring is provided in Table 1.

In Turkey, there are 26,600 FMP units, with each unit using 1 FMP at a time. As of September 18, 2023, FMPs working at 26,210 (98.5%) unique FMP units have logged into the DMP at least once, and 22,982 (86.4%) FMP units have performed at least 1 encounter.

Table 2 details the nationwide coverage rates per disease module and encounter type as of September 18, 2023. It includes the cumulative target population size and the unique number of patients screened or monitored at least once. During this period, DMP screenings led to new diagnoses: 144,768 for hypertension, 490,346 for diabetes, 534,423 for high cardiovascular risk, and 3,545,573 for obesity. These individuals were diagnosed with these chronic diseases for the first time, following evidence-based clinical guidelines.

**Figure 6:**
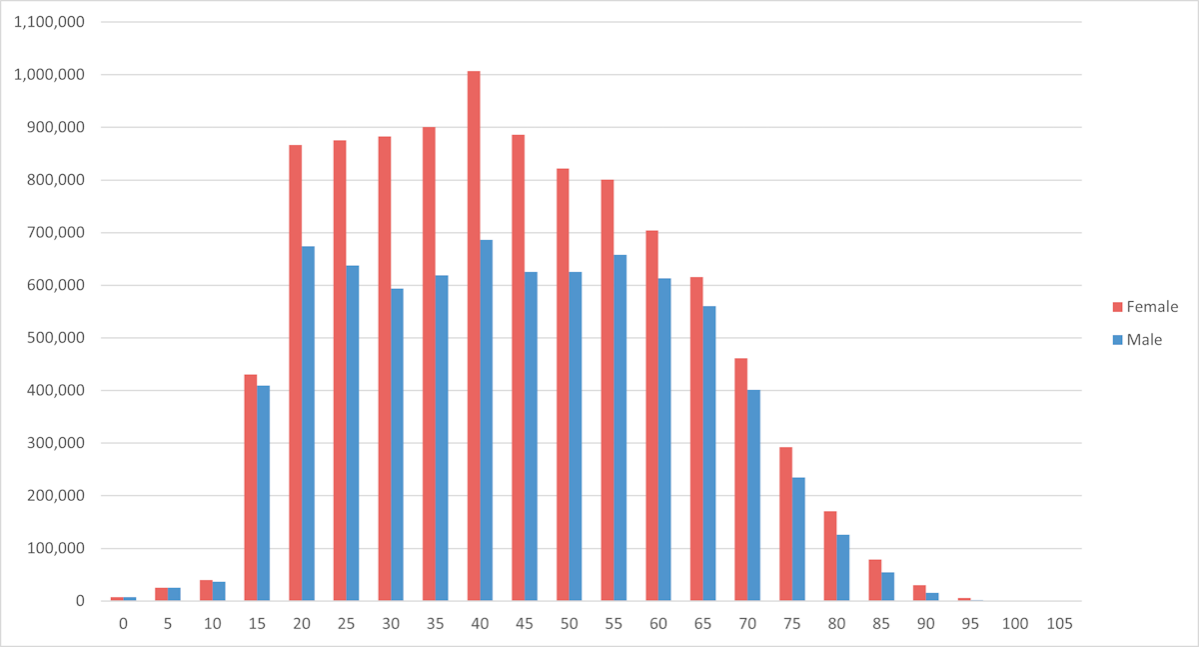
Age histograms of disease management platform patients: female on the left and male on the right.

Age histograms of DMP patients who have been screened or monitored at least once are provided per sex in Figure 6.

**Figure 7:**
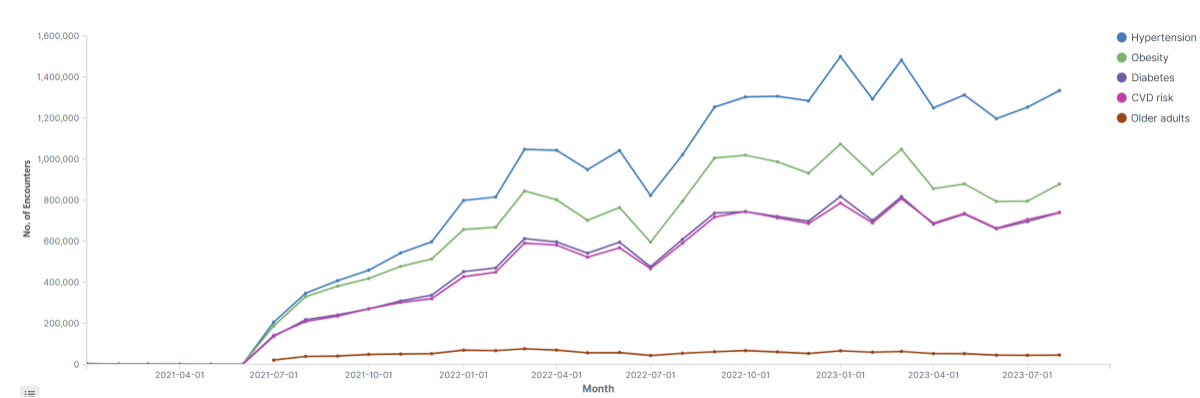
Disease management platform encounters per month by module. CVD: cardiovascular disease.

Piloting studies occurred from October to December 2019, and the system has been fully operational nationwide since January 2020. Use notably increased with FMP salary incentivization calculations on July 1, 2021 (Figure 7), showing monthly encounter numbers by module from the start of 2021. Since then, encounters have steadily risen, with minor drops during summer holidays, and the distribution among DMP modules has remained consistent.

**Figure 8:**
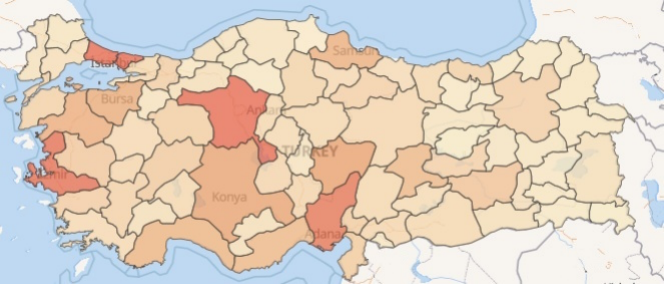
Encounters by city on a map.

Figure 8 displays the distribution of total DMP encounters per city in Turkey, with colors intensifying as encounter numbers rise. Although higher numbers generally align with city populations, outliers exist, as seen in the top 10 performing cities outlined in Table 3. Despite Istanbul having Turkey’s largest population, it only slightly surpasses Ankara in DMP encounters. This is mainly due to the high patient load per FMP in Istanbul. FMPs overseeing over 4000 citizens are exempt from DMP use due to their heavy workload. Table 3 also provides patient average age and encounter duration information.

The performance of the FMPs is assessed monthly. The cumulative targets and realized achievement rates for January 2023 are provided in Table 4. An achievement rate of 23.1% (4,508,841/19,546,041) for the entire population represents significant advancement compared with the 3.9% (511,198/13,117,900) achievement rate in July 2021.

The DMP system recommends personalized treatment goals such as systolic blood pressure, low-density lipoprotein cholesterol, and weight based on clinical guidelines. After a treatment goal is set, the DMP also assesses progress toward the goal in subsequent encounters. As of September 18, 2023, approximately 12.4 million of these treatment goals have been assessed, and the achievement rates are presented in Table 5. These assessments provide valuable information for FMPs caring for their patients.

At present, the performance of the FMPs is quantitatively calculated based on the number of performed encounters. However, the MOH envisions transitioning to a qualitative performance evaluation in midterm, where treatment goals and their achievement rates will play a significant role.

The system is highly performant and scalable. On a selected working day, February 14, 2023, the onFHIR.io HL7 FHIR Repository handled a total of 105.7 million FHIR interactions with an average response time of 31.3 milliseconds. During peak times of the day, the system can effortlessly manage up to 5000 FHIR interactions per second. Multimedia Appendix 2 illustrates the distribution and average response time of FHIR interactions on this day.

Among all FHIR requests, 57.4% (60.7 million) are search interactions, which are extensively used by the DMP web app to find, display, and forward specific clinical concept values to CDSS. Following search interactions, update interactions make up 33.2% (35.1 million) of the requests and are also used for resource creation when a provided resource ID is available. The average response times for read and search interactions are only 3.9 and 6.4 milliseconds, respectively. In the case of transactions and batch interactions, the average response times are even lower than update interaction alone, thanks to the parallelization of contained requests within onFHIR.io. As of September 18, 2023, onFHIR.io maintains a repository of 16.3 billion FHIR resources, totaling 22.4 terabytes in size, including care planning data by DMP and EHR/PHR data synchronized from e-Nabiz.

**Table 1:** Total screening and monitoring encounters per module.

Module	Screening (n=45,166,536), n (%)	Monitoring (n=28,548,733), n (%)	Total (n=73,715,269), n (%)
Hypertension	13,857,594 (30.7)	12,046,449 (42.2)	25,904,043 (35.1)
Obesity	18,029,994 (39.9)	800,480 (2.8)	18,830,474 (25.5)
Diabetes	8,914,193 (19.7)	5,071,646 (17.8)^a^	13,985,839 (19.0)
CVD^b^ risk	4,364,755 (9.7)	9,182,814 (32.2)	13,547,569 (18.4)
Older adult	N/A^c^	1,447,344 (5.1)	1,447,344 (2.0)

^a^Only the patients monitored in primary care are listed; advanced obesity cases (a BMI over 40 kg/m^2^ or a BMI between 30 and 40 kg/m^2^ supported with additional comorbidities) are monitored in secondary and tertiary care.

^b^CVD: cardiovascular disease.

^c^N/A: not applicable.

**Table 2:** Coverage rate of citizens in target population lists.

Module and encounter type			All citizens in target population, n		Screened and monitored patients, n		Coverage rate (%)
**Hypertension**							
	Screening	48,443,467		10,820,774		22.3	
	Monitoring	14,943,378		4,083,057		27.3	
**Obesity**							
	Screening	59,956,288		14,640,013		24.4	
	Monitoring	769,654^a^		383,920		49.9	
**Diabetes**							
	Screening	27,450,172		6,486,947		23.6	
	Monitoring	7,588,543		2,472,585		32.6	
**CVD^b^ risk**							
	Screening	17,276,617		3,319,070		19.2	
	Monitoring	17,759,500		5,078,665		28.6	
**Older adult**							
	Monitoring	8,770,474		1,056,766		12.0	

^a^Only those in the primary care obesity monitoring list, as explained in Table 1.

^b^CVD: cardiovascular disease.

**Table 3:** Top 10 performing cities.

City	Total population^a^	Rank^b^	Number of encounters	Number of patients	Average age of patients (years)	Average duration (minutes)
Istanbul	15,907,951	1	4,974,972	1,286,640	50.5	1.21
Ankara	5,782,285	2	4,918,745	1,084,797	51.7	1.13
Izmir	4,462,056	3	4,395,653	937,607	53.4	1.18
Adana	2,274,106	7	3,531,441	709,075	51.3	0.99
Kayseri	1,441,523	15	2,633,349	479,849	51.7	1.06
Antalya	2,688,004	5	2,617,274	636,535	51.4	1.08
Konya	2,296,347	6	2,607,459	579,927	51.2	1.15
Bursa	3,194,720	4	2,408,817	525,016	52.3	1.18
Balikesir	1,257,590	17	2,322,111	425,171	55.7	1.13
Samsun	1,368,488	16	2,237,385	422,438	54.2	1.06

^a^2022 census data by the Turkish Statistical Institute (TurkStat).

^b^The rank of cities in Turkey by total population count.

**Table 4:** Screening and monitoring encounters per module in January 2023.

Module and encounter type		Monthly target	Number of encounters	Achievement rate (%)
**Hypertension**				
	Screening	3,868,662	789,699	20.4
	Monitoring	4,795,117	709,004	14.8
**Obesity**				
	Screening	4,849,306	1,083,414	22.3
	Monitoring	53,770	51,512	95.8
**Diabetes**				
	Screening	898,665	670,502	74.6
	Monitoring	2,128,955	279,071	13.1
**CVD^a^ risk**				
	Screening	742,634	262,419	35.3
	Monitoring	1,488,004	589,523	39.6
**Older adult**				
	Monitoring	720,928	73,697	10.2
Total		19,546,041	4,508,841	23.1

^a^CVD: cardiovascular disease.

**Table 5:** Achievement rates of treatment goals.

Treatment goal	Achievement rate (%)
Systolic BP^a^	88.8
Diastolic BP	94.2
Fasting glucose	52.0
HbA_1c_^b^	61.5
LDL^c^ cholesterol	14.8
HDL^d^ cholesterol	63.2
Triglyceride	52.6
Weight	5.6
BMI	6.3
Waist circumference	2.9

^a^BP: blood pressure.

^b^HbA_1c_: hemoglobin A_1c_.

^c^LDL: low-density lipoprotein.

^d^HDL: high-density lipoprotein.

## Discussion

### Principle Findings and Lessons Learned

We have demonstrated that as of September 18, 2023, the DMP has been used by more than 25,000 users to conduct over 73 million screening and monitoring encounters for more than 16 million individuals. The national directive incentivizing FMPs to conduct screening and monitoring for chronic diseases is one of the contributing factors to this success.

We demonstrated the platform’s efficient horizontal scalability, serving thousands of HCPs daily without performance issues. DMP screenings identified approximately 150,000 new hypertension cases, over 490,000 diabetes cases, more than 500,000 high cardiovascular risk cases, and over 3.5 million obesity cases. This allowed timely treatment in line with evidence-based guidelines.

We have shown that the system seamlessly interoperates with existing national EHR via HL7 FHIR. It enables accessing and processing patient data from various sources to provide personalized care plan guidance, maximizing the effectiveness of evidence-based decision support services. The DMP has achieved all 5 levels of the 5S Model as proposed by Haynes [44] for the successful implementation of information services for evidence-based health care decisions. Continuous cocreation activity involving members of the Turkish MOH has contributed this success, along with the interoperability architecture based on international standards. On the other hand, we have collected feedback from FMPs to encourage us to also enable seamless integration with the national e-Prescription and national appointment system. FMPs need to manually input prescription and appointment recommendations into the other systems. Future plans include integrating these national systems directly to the DMP as well.

Although we have demonstrated that, through a repeatable and well-defined cocreation methodology, the system can be easily extended to address additional diseases, it still requires implementation effort from developers. We plan to extend the DMP system with administrative interfaces. This will enable subject matter experts from the MOH to create new disease screening and monitoring modules using form-based design interfaces.

Finally, although FMPs conduct screening and monitoring, specialists can view patient dashboards but cannot perform encounters; this can be easily enabled with the DMP’s role-based access control mechanism, pending organizational decisions for national-scale implementation.

The system is operated as a part of national health IT ecosystem funded by the budget of the Turkish MOH. Open-source technologies have been used; hence, additional licensing fee has not incurred. Approximately 80% of the budget is spent for software development, 15% for project management, and 5% for training costs. Initial development phase has lasted 2 years. In the last 2.5 years, the system is under maintenance, and new disease modules have been developed.

### Prospective Benefits and Impact

The system paves the way forward value-based care, where patient outcomes are monitored, and providers are incentivized for improving health. Currently, the DMP sets individual clinical goals (eg, HbA_1c_ and BMI) based on evidence-based guidelines. It monitors FMP performance in achieving these targets through close screening and monitoring. FMPs are presently incentivized based on screening and monitoring visits, but the system is ready to adopt value-based care by monitoring clinical targets.

DMP implementation opens opportunities to collect real-time research data, measuring the effectiveness of nationwide disease management protocols. Continuously gathering information about patients’ disease status and recording outcomes from screening and monitoring visits, the generated data provide valuable insights into disease management.

### Conclusions

This paper introduces a nationwide DMP designed for effective chronic disease screening and management, aligning with evidence-based clinical guidelines to enhance health care quality. With its user-friendly interfaces, it guides FMPs through personalized care planning with checklists for medication orders, referrals, lab tests, and risk screening. The system has been operational nationwide since January 2020. We have demonstrated seamless EHR integration, scalability, performance, and effectiveness in early diagnosis and meeting clinical targets. Future work includes a comprehensive study to analyze the direct clinical and cost-saving effects of the DMP on chronic disease management in Turkey.

## Appendix

Multimedia Appendix 1 Eligibility criteria for target populations for screening and monitoring encounters.

Multimedia Appendix 2 FHIR interactions in a day: request count on the left and average response time on the right. FHIR: Fast Healthcare Interoperability Resources.

## Abbreviations

CDSS: clinical decision support service
DMP: Disease Management Platform
EHR: electronic health record
FHIR: Fast Healthcare Interoperability Resources
FMP: family medicine practitioner
HbA_1c_: hemoglobin A_1c_
HCP: health care professional
HL7: Health Level Seven
ICD-10: International Classification of Diseases, Tenth Revision
MOH: Ministry of Health
PHR: personal health record

## Data Availability

The data sets generated and/or analyzed during this study are available from the corresponding author on reasonable request.

## References

[1] (2020). Life tables, 2017-2019. Turkish Statistical Institute.

[2] Non-communicable diseases and risk factors cohort study for Turkey. Republic of Turkey Ministry of Health.

[3] Yucesan M, Gul M, Mete S, Celik E, Bouchemal N (2019). A forecasting model for patient arrivals of an emergency department in healthcare management systems. Intelligent Systems for Healthcare Management and Delivery.

[4] Omid P, Bilal A, Despotou G, Keung SNLC, Mohamad Y, Gappa H, Erturkmen GBL, Yuksel M, Gencturk M, Schmidt-Barzynski W, Steinhoff A, Robbins T, Kyrou I, Randeva H, Ayadi J, Arvanitis TN, Córcoles RA, Abizanda P, Le K, Jiménez EG, Céspedes AA, Kaba E, Muir H (2023). CAREPATH methodology for development of computer interpretable, integrated clinical guidelines.

[5] (2019). Recommendations on digital interventions for health system strengthening–research considerations. World Health Organization.

[6] Agarwal S, Glenton C, Tamrat T, Henschke N, Maayan N, Fønhus MS, Mehl GL, Lewin S (2021). Decision-support tools via mobile devices to improve quality of care in primary healthcare settings. Cochrane Database Syst Rev.

[7] Kawamoto K, Houlihan CA, Balas EA, Lobach DF (2005). Improving clinical practice using clinical decision support systems: a systematic review of trials to identify features critical to success. BMJ.

[8] Bright TJ, Wong A, Dhurjati R, Bristow E, Bastian L, Coeytaux RR, Samsa G, Hasselblad V, Williams JW, Musty MD, Wing L, Kendrick AS, Sanders GD, Lobach D (2012). Effect of clinical decision-support systems: a systematic review. Ann Intern Med.

[9] Fortmann J, Lutz M, Spreckelsen C (2022). System for context-specific visualization of clinical practice guidelines (GuLiNav): concept and software implementation. JMIR Form Res.

[10] Garg AX, Adhikari NKJ, McDonald H, Rosas-Arellano MP, Devereaux PJ, Beyene J, Sam J, Haynes RB (2005). Effects of computerized clinical decision support systems on practitioner performance and patient outcomes: a systematic review. JAMA.

[11] Shiffman RN, Liaw Y, Brandt CA, Corb GJ (1999). Computer-based guideline implementation systems: a systematic review of functionality and effectiveness. J Am Med Inform Assoc.

[12] Poller L, Shiach CR, MacCallum PK, Johansen AM, Münster AM, Magalhães A, Jespersen J (1998). Multicentre randomised study of computerised anticoagulant dosage. European concerted action on anticoagulation. Lancet.

[13] Samore MH, Bateman K, Alder SC, Hannah E, Donnelly S, Stoddard GJ, Haddadin B, Rubin MA, Williamson J, Stults B, Rupper R, Stevenson K (2005). Clinical decision support and appropriateness of antimicrobial prescribing: a randomized trial. JAMA.

[14] Goud R, de Keizer NF, ter Riet G, Wyatt JC, Hasman A, Hellemans IM, Peek N (2009). Effect of guideline based computerised decision support on decision making of multidisciplinary teams: cluster randomised trial in cardiac rehabilitation. BMJ.

[15] Filippi A, Sabatini A, Badioli L, Samani F, Mazzaglia G, Catapano A, Cricelli C (2003). Effects of an automated electronic reminder in changing the antiplatelet drug-prescribing behavior among Italian general practitioners in diabetic patients: an intervention trial. Diabetes Care.

[16] Erturkmen GBL, Yuksel M, Sarigul B, Arvanitis TN, Lindman P, Chen R, Zhao L, Sadou E, Bouaud J, Traore L, Teoman A, Keung SNLC, Despotou G, de Manuel E, Verdoy D, de Blas A, Gonzalez N, Lilja M, von Tottleben M, Beach M, Marguerie C, Klein GO, Kalra D (2019). A collaborative platform for management of chronic diseases via guideline-driven individualized care plans. Comput Struct Biotechnol J.

[17] von Tottleben M, Grinyer K, Arfa A, Traore L, Verdoy D, Keung SNLC, Larranaga I, Jaulent MC, De Manuel Keenoy E, Lilja M, Beach M, Marguerie C, Yuksel M, Erturkmen GBL, Klein GO, Lindman P, Mar J, Kalra D, Arvanitis TN (2022). An integrated care platform system (C3-Cloud) for care planning, decision support, and empowerment of patients with multimorbidity: protocol for a technology trial. JMIR Res Protoc.

[18] Lobach DF, Hammond WE (1997). Computerized decision support based on a clinical practice guideline improves compliance with care standards. Am J Med.

[19] Dexter PR, Perkins S, Overhage JM, Maharry K, Kohler RB, McDonald CJ (2001). A computerized reminder system to increase the use of preventive care for hospitalized patients. N Engl J Med.

[20] Wang Z, An J, Lin H, Zhou J, Liu F, Chen J, Duan H, Deng N (2021). Pathway-driven coordinated telehealth system for management of patients with single or multiple chronic diseases in China: system development and retrospective study. JMIR Med Inform.

[21] Ramirez M, Chen K, Follett RW, Mangione CM, Moreno G, Bell DS (2020). Impact of a "chart closure" hard stop alert on prescribing for elevated blood pressures among patients with diabetes: quasi-experimental study. JMIR Med Inform.

[22] Burack RC, Gimotty PA, Simon M, Moncrease A, Dews P (2003). The effect of adding Pap smear information to a mammography reminder system in an HMO: results of randomized controlled trial. Prev Med.

[23] McPhee SJ, Bird JA, Fordham D, Rodnick JE, Osborn EH (1991). Promoting cancer prevention activities by primary care physicians. Results of a randomized, controlled trial. JAMA.

[24] Bates DW, Kuperman GJ, Rittenberg E, Teich JM, Fiskio J, Ma'luf N, Onderdonk A, Wybenga D, Winkelman J, Brennan TA, Komaroff AL, Tanasijevic M (1999). A randomized trial of a computer-based intervention to reduce utilization of redundant laboratory tests. Am J Med.

[25] Overhage JM, Tierney WM, Zhou XH, McDonald CJ (1997). A randomized trial of "corollary orders" to prevent errors of omission. J Am Med Inform Assoc.

[26] Wasylewicz ATM, Scheepers-Hoeks AMJW, Kubben P, Dumontier M, Dekker A (2019). Clinical decision support systems. Fundamentals of Clinical Data Science.

[27] Stagg BC, Stein JD, Medeiros FA, Wirostko B, Crandall A, Hartnett ME, Cummins M, Morris A, Hess R, Kawamoto K (2021). Special commentary: using clinical decision support systems to bring predictive models to the glaucoma clinic. Ophthalmol Glaucoma.

[28] Sim I, Gorman P, Greenes RA, Haynes RB, Kaplan B, Lehmann H, Tang PC (2001). Clinical decision support systems for the practice of evidence-based medicine. J Am Med Inform Assoc.

[29] (2018). Classification of digital health interventions v1.0: a shared language to describe the uses of digital technology for health. World Health Organization.

[30] Franck CP, Babington-Ashaye A, Dietrich D, Bediang G, Veltsos P, Gupta PP, Juech C, Kadam R, Collin M, Setian L, Pons JS, Kwankam SY, Garrette B, Barbe S, Bagayoko CO, Mehl G, Lovis C, Geissbuhler A (2023). iCHECK-DH: guidelines and checklist for the reporting on digital health implementations. J Med Internet Res.

[31] Welcome to FHIR. HL7 FHIR Release 4.

[32] HL7 FHIR Accelerator™ Program. HL7 International.

[33] What is FHIR?. The Office of the National Coordinator for Health Information Technology.

[34] (2018). Heat wave: the U.S. is poised to catch FHIR in 2019. HealthITBuzz.

[35] Ayaz M, Pasha MF, Alzahrani MY, Budiarto R, Stiawan D (2021). The Fast Health Interoperability Resources (FHIR) standard: systematic literature review of implementations, applications, challenges and opportunities. JMIR Med Inform.

[36] Vorisek CN, Lehne M, Klopfenstein SAI, Mayer PJ, Bartschke A, Haese T, Thun S (2022). Fast Healthcare Interoperability Resources (FHIR) for interoperability in health research: systematic review. JMIR Med Inform.

[37] (2022). FHIR (Fast Healthcare Interoperability Resources). NHS Digital.

[38] (2019). Medical Informatics Initiative demonstrates ability to standardise healthcare data across Germany according to FHIR. Medical Informatics Initiative Germany.

[39] HL7 FHIR® based secure data repository. onFHIR.io.

[40] SMART App launch: scopes and launch context. HL7 International.

[41] Birinci S, Kacır MF, Seker M, Dogrul M (2022). National Healthcare Technology Initiative. National Technology Initiative.

[42] USS Services.

[43] HL7 CDS hooks. HL7 International.

[44] Haynes RB (2006). Of studies, syntheses, synopses, summaries, and systems: the "5S" evolution of information services for evidence-based healthcare decisions. Evid Based Med.

