# Supplementary material for: A Nationwide Chronic Disease Management Solution via Clinical Decision Support Services: Software Development and Real-Life Implementation Report
Source: JMIR Med Inform. 2024 Jan 19;12:e49986. doi: 10.2196/49986 (PMC10837759; doi:10.2196/49986)
Supplement: Multimedia Appendix 1 [file medinform_v12i1e49986_app1.docx]

**Multimedia Appendix 1**

**Table S1.** Eligibility criteria for target populations for screening and monitoring.

| **Eligibility criteria for Diabetes Screening** | All men and women over the age of 40 (including the age of 40) from the strictly registered population affiliated with the family medicine unit who are not diagnosed with diabetes via ICD-10 E10-E14 codes and subcodes, or who do not use antidiabetic drugs (ATC Code: A10 - Antidiabetic Drugs) even if they are diagnosed, including all subcodes of ICD-10 E10-E14.  Screening should be performed once every three years (or more frequently according to the physician's risk assessment). |
| --- | --- |
| **Eligibility criteria for Hypertension Screening** | All men and women over the age of 18 (including the age of 18) from the strictly registered population affiliated with the family medicine unit who are not diagnosed with hypertension via ICD I10-I15 codes and subcodes and ICD I25.0, I25.1, I25.8 and I25.9 codes, or who do not use antihypertensive drugs even if diagnosed (ATC Code: C02 - Antihypertensive Drugs, C03 - Diuretic Drugs - Urinary Enhancing Drugs, C07 - Beta Blocking Agents, C08 - Calcium Channel Blockers, C09 - Blood Pressure Regulating Drugs) contact list. Screening should be performed once every two years (or more frequently according to the physician's risk assessment). |
| **Eligibility criteria for CVD Risk Screening** | All men and women over the age of 40 (including the age of 40) from the strictly registered population affiliated with the family medicine unit who are not diagnosed with cardiovascular disease via ICD-10 E78, I10-I15 codes and subcodes, or who do not use Antihyperlipidemic (ATC Code: C10 - Lipid Metabolism Drugs) even if they are diagnosed, including all subcodes of ICD-10 E78, I10-I15.  Screening should be performed once every two years (or more frequently according to the physician's risk assessment). |
| **Eligibility criteria for Obesity Screening** | All men and women over the age of 18 (including the age of 18) from the strictly registered population affiliated with the family medicine unit who are not diagnosed with obesity via DCR.^a^ |
| **Eligibility criteria for Diabetes Monitoring** | List of people diagnosed with diabetes via ICD E10-E14 codes and all subcodes and using antidiabetic drugs (ATC Code: A10 - Antidiabetic Drugs) within the population affiliated with Family Medicine. |
| **Eligibility criteria for Hypertension Monitoring** | List of people diagnosed with diabetes via ICD I10-I15 codes and subcodes and ICD I25.0, I25.1, I25.8 and I25.9 codes and using antihypertensive drugs (ATC Code: C02 - Antihypertensive Drugs, C03 - Diuretic Drugs - Urinary Enhancing Drugs, C07 - Beta Blocking Agents, C08 - Calcium Channel Blockers, C09 - Blood Pressure Regulating Drugs) within the population affiliated with Family Medicine. |
| **Eligibility criteria for CVD Risk Monitoring** | List of people diagnosed with the following codes:   - I10-I11-I12-I13-I15 codes and subcodes - I20-I21-I22-I23-I24-I25 codes and subcodes - I50-I51 codes and subcodes - I60-I61-I62 codes and subcodes - I63-I64-I65-I66 codes and subcodes - I67-I68-I69-G45-G46 codes and subcodes - I70 code and subcodes - I71 code and subcodes - I72 code and subcodes - I73 code and subcodes - I74 code and subcodes - E10-E14 codes and subcodes - E78 code and subcodes   and using antihyperlipidemic (ATC Code: C10 - Lipid Metabolism Drugs) within the population affiliated with Family Medicine. |
| **Eligibility criteria for Obesity Monitoring** | List of people diagnosed with obesity via DCR. |

^a^Although obesity prevalence is quite high in Turkey (21.1% in adults [1]), the use of obesity ICD-10 codes (E66) was not common in clinical records and BMI related measurements were not considered to be recent and reliable; hence obesity monitoring list was composed of only ~51k people while almost the whole 18+ population was put into the screening list at T0.

Reference

1. Turkish Statistical Institute. Turkish Health Research, 2019 [accessed January 6, 2024] <https://data.tuik.gov.tr/Bulten/Index?p=Turkiye-Saglik-Arastirmasi-2019-33661>
