# Supplementary figures and images for: A Nationwide Chronic Disease Management Solution via Clinical Decision Support Services: Software Development and Real-Life Implementation Report
Source: JMIR Med Inform. 2024 Jan 19;12:e49986. doi: 10.2196/49986 (PMC10837759; doi:10.2196/49986)

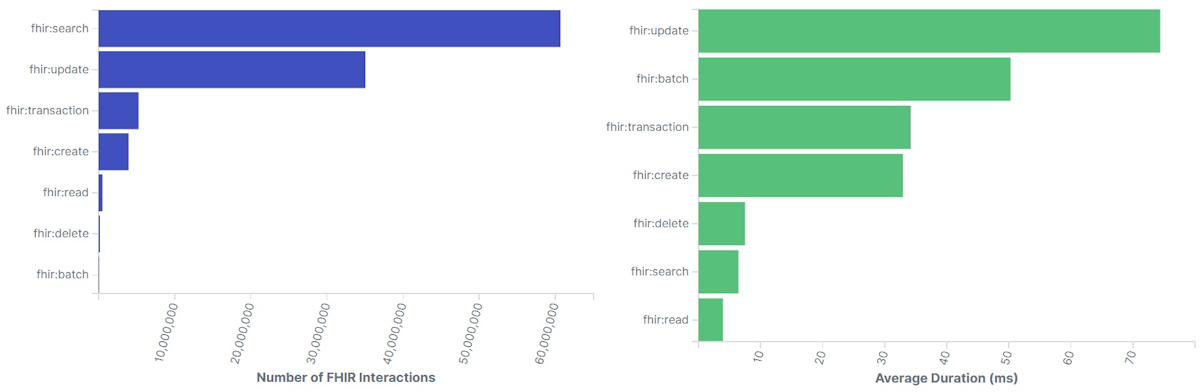

Supplement: Multimedia Appendix 2 [file medinform_v12i1e49986_app2.png]
